# Application of 3D-Bioprinting in Treatment of Chronic Wounds: A Review

**DOI:** 10.3390/life16040581

**Published:** 2026-04-01

**Authors:** Miroslava Chortova, Elean Zanzov, Vanya Anastasova

**Affiliations:** Department of Burns, Plastic and Reconstructive Surgery, Medical University-Plovdiv, 4100 Plovdiv, Bulgaria; elean.zanzov@mu-plovdiv.bg (E.Z.); vanya.anastasova@mu-plovdiv.bg (V.A.)

**Keywords:** 3-D bioprinting, chronic wounds, biomaterials, adipose tissue, mesenchymal stem cells, regenerative medicine

## Abstract

Chronic wounds represent a significant global healthcare challenge, affecting millions of patients and imposing substantial economic burdens on healthcare systems. Traditional wound management approaches often fail to address the complex pathophysiology underlying chronic wounds, including persistent inflammation, impaired angiogenesis, and disrupted extracellular matrix remodeling. Three-dimensional (3D) bioprinting has emerged as a transformative technology that enables the fabrication of patient-specific, biomimetic tissue constructs capable of addressing these intricate challenges. This comprehensive review synthesizes recent advances in 3D bioprinting for chronic wound treatment, examining bioprinting technologies, biomaterial innovations, mechanisms of wound healing, and clinical applications. Recent studies demonstrate that bioprinted constructs incorporating living cells, growth factors, and bioactive molecules can significantly accelerate wound closure, enhance vascularization, and restore functional skin architecture. Notable innovations include in situ bioprinting systems, photosynthetic scaffolds for oxygen delivery, and immunomodulatory bioinks. While significant technical challenges remain—including vascularization, scalability, and regulatory approval—the integration of advanced bioprinting techniques with regenerative medicine principles offers unprecedented opportunities for personalized chronic wound care and improved patient outcomes.

## 1. Introduction

Chronic wounds represent a significant and growing public health challenge, defined as lesions that fail to progress through the normal healing process within an expected timeframe—typically failing to heal within three months despite appropriate care [1,2]. These wounds encompass several distinct types, including diabetic foot ulcers (DFUs), pressure ulcers (PUs), and venous leg ulcers (VLUs), which collectively affect an estimated 1–2% of the population in developed countries [1,2]. Chronic wounds predominantly afflict medically complex and older populations with comorbidities such as diabetes, vascular disease, and frailty, necessitating multidisciplinary care and long-term resource utilization [2]. The pathophysiology of chronic wounds is multifactorial and complex, involving both local and systemic factors. These wounds are characterized by persistent inflammation, impaired cellular proliferation, insufficient angiogenesis, and abnormal extracellular matrix (ECM) remodeling. Local factors such as ischemia, infection, and mechanical stress interact with systemic conditions including diabetes mellitus, vascular diseases, and immunodeficiency to create a wound environment that resists healing [3].

Diabetic foot ulcers represent one of the most serious complications of diabetes mellitus, with millions of affected individuals and rising prevalence globally [4]. These ulcers are strongly associated with peripheral arterial disease and neuropathy; in a multicentric cohort, 74.8% of DFU patients had peripheral arterial disease and 14.5% had peripheral neuropathy, with a median HbA1c of approximately 9.9% [5]. The vulnerable population primarily consists of older adults with diabetes and multiple comorbidities. In a review of Lo et al., 97.2% of neuro-ischemic ulcer patients were diabetic with high comorbidity burden and frailty [2]. Pressure ulcers occur predominantly in immobilized and institutionalized populations, with incidence rates varying significantly by care setting. In tertiary care settings, pressure injury incidence has been reported as high as 64.6 per 1000 inpatient admissions [3], while community primary care point prevalence was considerably lower at 0.03% [4]. The primary risk factors include immobility, advanced age, malnutrition, and sensory impairment [2]. Vulnerable groups include hospitalized patients, long-term care residents, and those with limited mobility. Venous leg ulcers, primarily caused by chronic venous insufficiency, account for approximately 80% of chronic lower-leg ulcers [6]. Community primary care prevalence has been reported at 0.04% [7], with prevalence increasing with age. These ulcers are predominantly managed in outpatient specialist settings and affect older adults with chronic venous disease [2,8]. The pathophysiology of VLUs centers on factors that impair venous return and chronic venous insufficiency.

The clinical burden of chronic wounds is substantial, characterized by prolonged healing times, high complication rates, and significant recurrence. Average length of stay for wound episodes has been documented at 17.7 days—approximately 2.4 times longer than average acute admissions [5]. For diabetic foot ulcers specifically, hospitalization length varies dramatically by severity: ulcer-only admissions average 13.3 days, minor amputation admissions average 20.5 days, and major amputation admissions extend to 59.6 days [4]. Complications are frequent and severe. In one large DFU cohort of 1729 patients, amputation distribution included toe amputation in 36.4%, transmetatarsal amputation in 16.9%, and major amputation in 6.5% of cases [6]. Recurrence rates are particularly concerning for venous leg ulcers, with 1-year recurrence rates of approximately 52.5% and a median time from healing to recurrence of 9.5 months in specialist outpatient populations [5]. Infection and readmission trends are rising, with substantial increases in 30-day readmissions for surgical site and wound-related problems [4].

The economic impact of chronic wounds on healthcare systems is staggering and increasing, with annual healthcare costs exceeding $25 billion in the United States [2,5]. Per-episode charges in tertiary care settings have been documented at USD $12,967, with one healthcare cluster estimating total inpatient wound costs at USD $216 million in 2017 alone [2]. Chronic wound management has been estimated to consume approximately 2–4% of health budgets in some Western countries, representing major system pressure [7]. The wound-care products market alone has been projected in the multi-billion-dollar range, reflecting sustained high demand for dressings, devices, and advanced therapies [5]. Importantly, there has been a notable shift in service settings, with reductions in hospital outpatient expenditures and increases in care delivered through physician offices and durable medical equipment [4,6], necessitating care-pathway redesign and enhanced care coordination.

Beyond the clinical and economic burdens, chronic wounds substantially impair patients’ quality of life through multiple mechanisms. Patients frequently report pain, reduced mobility, social isolation, odor and exudate problems, and mental health sequelae [4]. In some surveys, diabetic patients have rated chronic wounds as having more dramatic quality-of-life effects than other major diabetes complications [4,6]. The rising burden of chronic wounds poses significant operational challenges for healthcare systems. Care fragmentation and shifts in service settings require care-pathway redesign and enhanced coordination. Additionally, there are substantial workforce and training gaps, with limited emphasis on chronic wound care in medical education despite the high public health impact [7].

Traditional wound management strategies—encompassing debridement, infection control, topical dressings, and surgical interventions—provide foundational care but often fail to address the complex molecular and cellular disruptions underlying chronic wounds. Advanced therapies, including growth factor supplementation, stem cell treatments, and tissue-engineered skin substitutes, have shown promise but face limitations related to cost, scalability, and the inability to fully recapitulate the native skin architecture. Three-dimensional bioprinting has emerged as a revolutionary technology capable of fabricating complex, patient-specific tissue constructs by precisely depositing cells, biomaterials, and bioactive molecules in defined spatial arrangements [2,4]. Unlike conventional tissue engineering approaches, 3D bioprinting enables the creation of biomimetic structures that mimic the hierarchical organization of native skin, including the epidermis, dermis, and vascular networks [3]. Recent advances in bioprinting technologies, biomaterial development, and our understanding of wound-healing mechanisms have positioned 3D bioprinting as a promising solution for chronic wound management.

This systematic review analyzes literature on the application of 3D bioprinting in chronic wound treatment published mainly from 2020 to 2026. The literature search included the following databases: Scopus, Web of Science, Google Scholar, and PubMed. The structure of the paper follows the Preferred Reporting Items for Systematic Reviews and Meta-Analyses (PRISMA) guidelines. We examine the diverse bioprinting technologies and techniques employed, analyze the biomaterials and bioinks developed for wound-healing applications, elucidate the mechanisms by which bioprinted constructs promote healing, and evaluate clinical outcomes from preclinical and clinical studies. We discuss the technical and translational challenges that must be addressed to realize the full clinical potential of this transformative technology. Additionally, we present our experience with Inkjet 3D-Bioprinting technology for treatment of DFUs. Three-dimensional bioprinting represents a promising frontier in addressing chronic wounds challenges through the creation of biomimetic tissue constructs that can address the complex molecular and cellular disruptions underlying chronic wounds.

## 2. Bioprinting Technologies and Techniques

The selection of bioprinting technology significantly influences the structural fidelity, cellular viability, and functional properties of bioprinted constructs. Recent studies have employed diverse bioprinting approaches, each offering distinct advantages for chronic wound applications.

### 2.1. Bioprinting Technologies, Based on Deposition Mechanism

Inkjet bioprinting releases droplets at high speed with good resolution. It requires low-viscosity bioinks and can stress cells from thermal/piezo pulses. Extrusion forces continuous filaments, handling high-viscosity, cell-dense bioinks and scaffolds but with lower resolution. Laser-assisted bioprinting is nozzle-free, offers high precision and good cell viability for delicate patterns but is complex and costly. Stereolithography creates very high-resolution parts from photopolymerizable bioinks and requires light-sensitive materials.

#### 2.1.1. Extrusion-Based and Inkjet Bioprinting

Extrusion-based bioprinting remains the most commonly used technique for wound-healing applications because it is versatile, cost-effective, and compatible with high-viscosity bioinks [8]. The approach uses pneumatic or mechanical forces to extrude bioinks through a nozzle, building constructs layer by layer. Recent reports show extrusion bioprinting can successfully produce diabetic wound dressings and skin substitutes. Double-crosslinked, angiogenic alginate/chondroitin sulfate patches created by extrusion bioprinting have been shown to enhance angiogenesis and extracellular matrix remodeling in diabetic wound models [9]. Likewise, polycaprolactone (PCL) scaffolds printed with levofloxacin on a Bio-X bioprinter provided sustained antibiotic release over four weeks and demonstrated antibacterial efficacy against Staphylococcus aureus and Escherichia coli; these scaffolds also displayed robust mechanical properties, with tensile stiffness of 25.30 N/mm for square designs, supporting the flexibility needed for wound applications [10].

**Figure 1:**
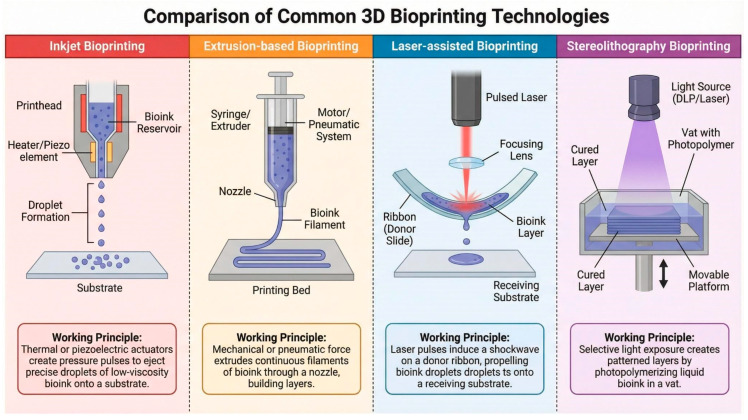
Most common types of 3D-Bioprinting deposition technologies.

Advanced dressings produced by extrusion bioprinting that incorporated decellularized adipose matrix, plasma, and human dermal fibroblasts maintained high cell viability for 11 days and released wound-healing cytokines—including interleukin-8 (IL-8), monocyte chemoattractant protein-1 (MCP-1), vascular endothelial growth factor (VEGF), and hepatocyte growth factor (HGF)—with MCP-1 levels rising up to 70-fold by day 11, indicating an ability to establish favorable molecular microenvironments for chronic wound repair [11] (Figure 1). Extrusion bioprinting combined with dual-photo source crosslinking is able to create human-derived skin organoids composed of keratinocytes, fibroblasts, and endothelial cells. When applied to full-thickness skin defects in immunodeficient mice, these customized organoids significantly accelerated healing by promoting in situ regeneration, epithelialization, vascularization, and reducing inflammation [12]. Inkjet bioprinting, in contrast to extrusion-based methods, delivers biomaterial as small, evenly spaced droplets, differing primarily in the mode of material deposition [13].

#### 2.1.2. Laser-Assisted Bioprinting

Laser-assisted bioprinting (LAB) is a nozzle-free technique that utilizes focused laser pulses to propel cell-laden droplets onto a substrate with extremely high resolution and precision [14]. Recent studies from 2020 to 2025 highlight LAB’s unique ability to handle viscous bio-inks and sensitive biological materials without the shear stress typically associated with extrusion-based methods, resulting in superior cell viability [15]. Advances in “absorber-free” laser bio-printing have been developed to eliminate the risk of metallic contamination from the absorbing layer, further enhancing the safety of printed tissues for clinical applications [16,17]. This technology has shown remarkable promise in printing high-cell-density constructs, making it ideal for fabricating complex tissues like skin models and corneal stroma where precise cell placement is critical [14,16]. Researchers are increasingly combining LAB with other bioprinting modalities to create multi-scale structures, leveraging laser precision for micro-patterning while using extrusion for bulk structural support [18]. Furthermore, recent papers demonstrate the efficacy of LAB in in situ bioprinting, where cells are deposited directly onto wound sites to accelerate healing and regeneration [19]. The high speed and non-contact nature of the process minimize contamination risks, positioning LAB as a powerful tool for next-generation tissue engineering and regenerative therapies [20] (Figure 1).

#### 2.1.3. Stereolithography Bioprinting

Stereolithography (SLA) 3D bioprinting has emerged as a high-precision technique for fabricating complex tissue constructs by using light to polymerize photosensitive bio-inks layer by layer [21]. SLA recent advancements have focused heavily on developing novel bio-resins that offer both high printability and excellent biocompatibility, overcoming historical limitations regarding cell viability [22]. Researchers have successfully utilized visible light-based SLA systems to reduce phototoxicity, allowing for the encapsulation of live cells within hydrogels like gelatin methacryloyl (GelMA) and polyethylene glycol diacrylate (PEGDA) with high survival rates [23]. This technology is particularly adept at creating microfluidic channels and vascular networks within tissue scaffolds, which are essential for nutrient delivery in thick tissues [23]. Furthermore, innovations in multi-material SLA have enabled the fabrication of heterogeneous structures that mimic the gradient properties of natural tissues, such as the bone-cartilage interface [23]. These developments position SLA as a leading method for applications in drug screening, disease modeling, and regenerative medicine [24] (Figure 1).

#### 2.1.4. Digital Light Processing (Dlp) Bioprinting

Digital light processing bioprinting offers superior resolution and printing speed compared to extrusion-based methods, enabling the fabrication of intricate microstructures with high cellular precision [24]. Fu et al. established a clinically translatable collagen-based DLP bioprinting platform using pro-angiogenic dual-crosslinked collagen bioinks for precise cell-laden printing [14]. This approach demonstrated enhanced vascular regeneration in angiogenesis-impaired diabetic wounds, addressing one of the critical challenges in chronic wound healing [14].

The high resolution achievable with DLP bioprinting (typically 10–100 μm) enables the creation of biomimetic microarchitectures that guide cellular behavior and tissue organization [15]. However, the limited range of photocrosslinkable bioinks and potential phototoxicity from UV exposure remain considerations for this technology [25].

Both SLA and DLP are vat photopolymerization techniques, meaning they use light to cure bioink into a solid structure. While they are very similar, the key difference lies in the light source and how they project light. SLA uses a laser, whereas DLP uses a digital projector screen. The laser creates the shape point by point, when DLP creates a whole layer simultaneously. That makes DLP generally faster which can lead to reduced environmental stress for the living cells and better survival [26].

**Figure 2:**
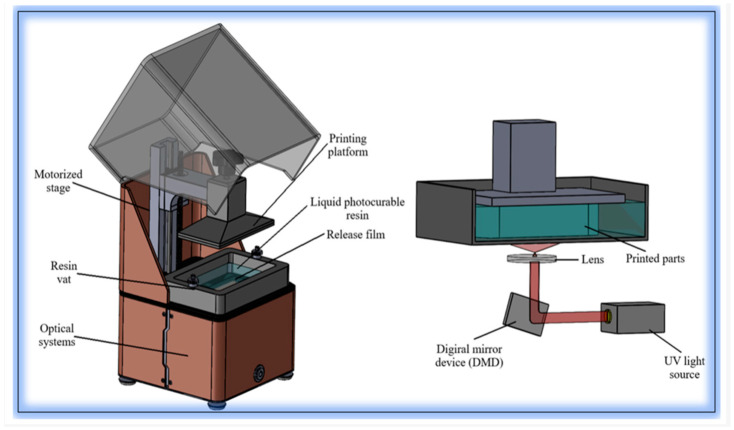
DLP working principle [25].

DLP bioprinting (Figure 2) mechanically projects a patterned image (via a DMD micromirror array) onto a thin layer of photopolymerizable bioink in a vat, curing that entire cross-section at once. After exposure the build platform shifts (up or down depending on bottom-up or top-down configuration) to remove the cured layer from the optical window and allow fresh resin to recoat the surface. In bottom-up systems a brief peel/separation step overcomes adhesion to the window before recoating; in top-down systems the platform simply moves to expose fresh resin. This cycle of image projection, polymerization, platform translation and resin replenishment repeats layer-by-layer until the part is finished [25].

### 2.2. Bioprinting Technologies, Based on Application Mechanism

#### 2.2.1. In Situ Bioprinting

In situ bioprinting represents a paradigm shift in wound treatment, enabling direct deposition of bioinks onto wound beds, thereby eliminating the need for ex vivo construct maturation and subsequent transplantation [27]. This approach offers several advantages, including improved conformity to irregular wound geometries, enhanced graft-host integration, and reduced handling-related cellular damage [28].

**Figure 3:**
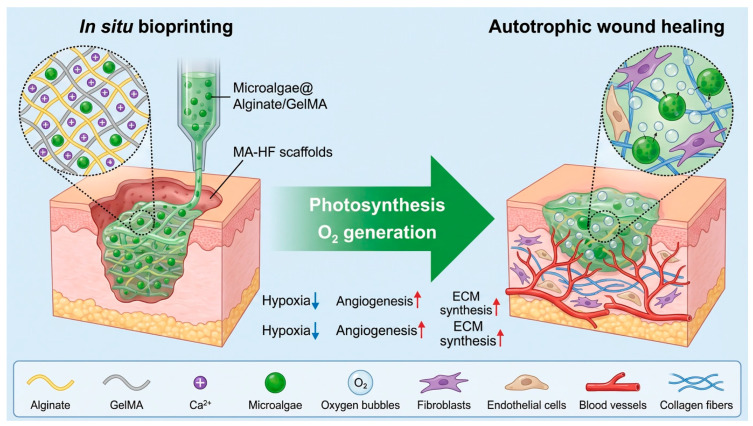
In situ Bioprinting process incorporating photosynthesising Microalgae.

Hypoxia is a major barrier to healing in diabetic wounds. Wang et al. introduced a groundbreaking approach by incorporating the microalga *Chlorella pyrenoidosa* into GelMA/alginate scaffolds [29]. Under light exposure, these algae perform photosynthesis, generating dissolved oxygen in situ. This oxygenation alleviates local hypoxia, reduces HIF-1α expression, and provides the metabolic fuel necessary for cell proliferation and angiogenesis. This represents an innovative in situ microfluidic-assisted 3D bioprinting strategy for depositing living photosynthetic scaffolds directly into diabetic wounds [29]. The system utilized a custom-made coaxial capillary microfluidic chip integrated with a programmable 3D printer, enabling the deposition of microalgae-laden hollow fibrous scaffolds at controlled flow rates (2 mL/h) and printing speeds (5 mm/s) [21]. The bioprinted scaffolds, composed of alginate (2.5% *w*/*v*) and gelatin methacrylate (GelMA, 5% *w*/*v*) incorporating *Chlorella pyrenoidosa* microalgae, produced sustainable oxygen under light conditions, alleviating hypoxia in diabetic wounds [28,29].

(Figure 3) Illustration of in situ 3D bioprinting living photosynthetic scaffolds for autotrophic wound healing. The microalgae-laden hollow fibrous (MA-HF) scaffolds can be directly printed in freeform wounds due to the rapid crosslinking between the Ca ions and alginate-based progels during a coaxial microfluidic printing process. After printing, the microalgae encapsulated in the MA-HF scaffolds serve as in situ autotrophic oxygen suppliers, which continuously generate oxygen under light illumination for enhanced wound healing by alleviating local hypoxia, accelerating angiogenesis, and promoting extracellular matrix (ECM) synthesis at wound sites [29].

In vivo studies demonstrated that the photosynthetic scaffolds achieved remarkable wound closure, with the light-exposed group reaching 1.1 ± 0.3% relative wound area by day 15, compared to 13.0 ± 1.5% for controls [29]. Additionally, CD31-positive microvessel densities increased to 15.6 ± 1.6% in treated wounds versus 5.5 ± 0.1% in controls, while hypoxia-inducible factor-1α (HIF-1α) expression decreased to 1.1 ± 0.2% compared to 13.2 ± 1.5% in controls. This autotrophic biosystem represents a novel approach to addressing hypoxia-related impairments in chronic wound healing. Xue et al. reviewed in situ bioprinting applications for tissue regeneration, highlighting studies where platelet-rich plasma-containing bioprinted constructs with dermal fibroblasts and epidermal stem cells were directly printed onto wound beds, leading to adequate reepithelization and faster wound closure within 21 days for both acute and chronic wounds [18,29].

In situ 3D bioprinting enables direct deposition of cell-laden bioinks into tissue defects during surgery, offering significant advantages over traditional pre-fabricated grafts across multiple tissue types. Practical applications have been demonstrated in skin wound repair, where robotic systems printed cell-loaded bioinks onto full-thickness defects achieving faster wound closure and more uniform re-epithelialization compared to commercial dressings [28]; craniofacial bone reconstruction, where autologous adipose-derived stem cell-laden bioinks printed into rabbit calvarial defects produced superior bone regeneration versus controls [30]; and pre-vascularized constructs, where endothelial cells printed in thermosensitive bioinks created functional vascular networks that enhanced angiogenesis-osteogenesis coupling in rat bone defects [31]. The key advantages of in situ bioprinting over pre-fabricated grafts include superior tissue integration through direct cell-tissue contact at the wound margin that enhances cellular communication and regeneration [32]; precise anatomical customization enabled by integrating 3D scanning and printing into a single intraoperative workflow, eliminating pre-fitting steps and reducing trial-and-error adjustments [29]; enhanced vascularization through spatial control of endothelial cell placement and creation of perfusable channels directly at the defect site [33]; streamlined surgical workflows that consolidate debridement, scanning, and printing into rapid point-of-care procedures [27]; and improved mechanical integration through accurate surface matching on complex anatomies, reducing micromotion and stress concentration [32]. Minimally invasive approaches using noninvasive NIR photopolymerization have even generated cartilaginous and muscle constructs in vivo without open surgery [30]. While challenges remain in equipment accessibility, bioink development, regulatory approval, and long-term clinical validation, preclinical evidence demonstrates that in situ bioprinting addresses fundamental limitations of pre-fabricated grafts and represents a paradigm shift toward personalized, intraoperative regenerative therapies [31].

#### 2.2.2. Coaxial and Microfluidic Bioprinting

Coaxial bioprinting facilitates the creation of core–shell structures capable of encapsulating cells, growth factors, or therapeutic agents within protective hydrogel shells, thereby offering controlled release and improved cellular protection [34]. This technique has been utilized to develop biphasic nanobioinks aimed at diabetic wound healing, effectively accelerating the process in type II diabetic mouse models by modulating angiogenesis and inflammation [35]. Furthermore, microfluidic systems have been integrated with coaxial 3D bioprinting to produce diabetic wound-healing dressings. This combination allows for precise control over bioink composition and spatial organization [35], making it possible to fabricate constructs with graded compositions that mimic the heterogeneous nature of native skin tissue [36].

## 3. Biomaterials for Wound Healing

The selection and formulation of biomaterials constitute critical determinants of bioprinted construct performance, influencing mechanical properties, cellular behavior, degradation kinetics, and therapeutic efficacy.

### 3.1. Natural Polymers

Natural polymers derived from biological sources offer inherent biocompatibility, biodegradability, and cell-recognition motifs that promote cellular adhesion, proliferation, and differentiation [25,37].

Alginate is a widely used natural polymer derived from brown algae, valued for its rapid gelation through ionic crosslinking with divalent cations such as calcium chloride. Alginate can be utilized in combination with chondroitin sulfate to create double-crosslinked angiogenic patches for diabetic wound healing. The alginate component provided structural integrity and printability, while chondroitin sulfate contributed to angiogenic signaling [38].

Gelatin and Gelatin Methacrylate (GelMA), both derived from collagen, are critical for cellular adhesion due to their retention of cell-binding RGD (Arg-Gly-Asp) sequences [28,39]. For instance, photosynthetic scaffolds have been engineered by combining GelMA (5% *w*/*v*) with alginate (2.5% *w*/*v*), utilizing lithium phenyl-2,4,6-trimethylbenzoylphosphinate (LAP, 0.1% *v*/*v*) as a photoinitiator for UV-induced crosslinking [21]. Furthermore, cell-adaptive hydrogels have been created from GelMA and sodium alginate, crosslinked via thermal, ionic, and photocuring techniques. These hydrogels incorporated shear-oriented polyethylene oxide (PEO) as a filler, generating anisotropic micropores that were found to enhance fibroblast-to-myofibroblast transition and accelerate wound closure [40].

Collagen, as the predominant protein in the extracellular matrix (ECM), is crucial for offering structural support and biochemical signals vital for tissue regeneration [33]. One application involved establishing a DLP bioprinting platform that utilized pro-angiogenic, dual-crosslinked collagen bioinks, which were shown to significantly improve vascular regeneration in diabetic wounds [41]. In another instance, methacrylated collagen (CMA) bioinks, at concentrations ranging from 10 to 50% (*w*/*w*) microfat, were successfully employed to sustain cell viability and metabolic activity for up to ten days [32].

Hyaluronic Acid (HA) is a glycosaminoglycan that plays crucial roles in wound healing, including regulation of inflammation, angiogenesis, and ECM organization [42]. The 3D-bioprinted peptide patches developed using GelMA/hyaluronic acid methacryloyl (HAMA) demonstrated excellent biocompatibility and angiogenesis, with the pro-angiogenic QHREDGS peptide covalently conjugated to extend its release [43].

### 3.2. Synthetic Polymers

Synthetic polymers offer tunable mechanical properties, controlled degradation rates, and reproducible manufacturing, though they typically lack inherent bioactivity [44].

Polycaprolactone (PCL) is a biodegradable polyester with excellent mechanical strength and slow degradation kinetics, making it suitable for load-bearing applications [45]. Glover et al. fabricated PCL scaffolds (MW 50,000) loaded with levofloxacin using extrusion bioprinting at 190 °C and 175 kPa, achieving sustained drug release for 4 weeks. The scaffolds provided mechanical support while delivering antibacterial therapy, addressing the dual challenges of structural integrity and infection control in diabetic wounds [46].

Polyethylene Oxide (PEO) serves as a sacrificial material to create porous structures. Shi et al. incorporated shear-oriented PEO filler into GelMA/sodium alginate hydrogels, creating anisotropic micropores that guided cellular organization and enhanced wound healing [29].

### 3.3. Composite and Hybrid Bioinks

Composite bioinks combine natural and synthetic polymers or incorporate inorganic materials to achieve synergistic properties that individual components cannot provide [36]. A pioneering approach integrated strontium silicate (SS) microcylinders into multicellular biomaterial inks using “cell-writing” bioprinting technology, where the SS microcylinders acted as stable, cell-induced angiogenic cues and the resulting bioprinted skin substitutes showed strong angiogenic activity in vitro and in vivo, markedly accelerating healing of acute and chronic wounds via improved graft–host integration and vascularized skin regeneration [37]. Composite bioinks were formulated by blending porcine decellularized adipose matrix (pDAM2) with alginate and plasma with pDAM2 supplying collagens, proteoglycans, glycoproteins, and associated proteins while plasma provided growth factors and cytokines; human dermal fibroblasts within these hydrogels maintained expression of fibronectin and collagen genes (COL1A1, COL1A2, COL3A1), establishing a favorable molecular microenvironment for wound healing [47]. Bioinspired 3D-printed scaffolds embedding DDAB-nano ZnO/nanofibrous microspheres were developed to promote regenerative diabetic wound healing, illustrating the potential of nanoparticle incorporation to boost antibacterial properties and support tissue regeneration [48].

### 3.4. Bioactive Components

The incorporation of cells, growth factors, and bioactive molecules transforms passive scaffolds into dynamic therapeutic systems capable of actively promoting wound healing [39].

#### 3.4.1. Cellular Components

Recent Research Has Shown the Incorporation of Various Cell Types Into Bioprinted Constructs, Such as Human Dermal Fibroblasts, Keratinocytes, Endothelial Cells, Mesenchymal Stem Cells, and Adipose-Derived Stromal Cells [11,12,22,32]. for Instance, One Study Successfully Developed Skin Organoids by Combining Keratinocytes, Fibroblasts, and Endothelial Cells, Which Then Formed Distinct Structures with Stromal Cores, Promoting in Situ Regeneration and Vascularization [49]. Another Investigation Illustrated That Multicellular Bioprinted Skin, When Composed of Multiple Cell Types, Was Capable of Forming Human-like Skin Architecture In Vivo [49].

Fibroblasts in bioprinted constructs provide dermal matrix production and mechanical remodeling that scaffold integration and re-epithelialization depend on, with their behavior including alignment, myofibroblast transition, and collagen/fibronectin deposition being strongly regulated by bioink composition, pore architecture, and mechanical cues [50]. In their role in healing, fibroblasts synthesize collagen I/III and fibronectin and drive granulation tissue formation and wound contraction, which supports epidermal ingrowth and tensile strength during repair [51]. Regarding scaffold interactions, fibroblasts respond to anisotropic micropores and aligned microarchitectures in GelMA/sodium alginate (SA)/PEO scaffolds by adopting oriented morphologies that promote myofibroblast transition and ECM remodeling [51]. The cellular mechanisms employed by fibroblasts include enhanced proliferation and spreading in cell-adaptive GelMA/PEO/SA hydrogels that present sparse, cell-friendly microenvironments [51], increased ECM production with elevated fibronectin and collagen deposition occurring within GelMA/nano-cellulose dermal formulations tuned for porosity and stiffness, and phenotype modulation where scaffold stiffness and anisotropy bias fibroblast-to-myofibroblast differentiation, affecting contraction and scar outcomes [52]. Fibroblast-laden dermal layers support angiogenesis indirectly by secreting proangiogenic ECM-bound factors and creating a permissive matrix, with 3D-printed dermis formulations containing fibroblasts correlating with greater neovascularization and improved wound contraction in mouse models [53]. In bilayer constructs, dermal fibroblasts promote endothelial network stability and keratinocyte maturation through ECM remodeling and paracrine signaling, with heterogeneous GelMA/nano-cellulose bioinks supporting dermal networks that enabled epidermal stratification in vitro. Therapeutic evidence from preclinical studies demonstrates that hydrogels with embedded fibroblasts accelerated full-thickness wound closure, increased collagen remodeling, and stimulated angiogenesis in rodent models [54], while gelatin–alginate/fibroblast dermal layers in bilayer grafts improved wound contraction and induced angiogenesis compared with controls in nude mouse wounds [55].

Endothelial cells establish perfusable microvasculature within bioprinted constructs and are essential for oxygen/nutrient supply, graft inosculation, and long-term survival, with scaffold design that includes channels, cores, or vascular fragments accelerating network formation and function [56]. In their role in healing, endothelial cells assemble into capillary-like networks that anastomose with host vasculature to perfuse grafts and sustain embedded cells [56]. Regarding scaffold interactions, pre-vascularized architectures such as core–shell extrusion (endothelial core, supportive GelMA shell) or sacrificial hollow channels enable endothelial tubulogenesis and lumen formation within printed constructs [57], while ECM composition with collagen I/fibronectin–based dermal bioinks supports EC self-assembly and rapid inosculation after implantation [57]. The cellular mechanisms employed by endothelial cells include vasculogenesis and tubulogenesis where ECs self-organize into interconnected microvascular networks inside collagen or GelMA matrices and form perfusable lumens in vivo [56,57], and paracrine signaling where ECs secrete and respond to VEGF and matrix cues, with inclusion of VEGF-mimetic peptides in GelMA patches enhancing EC proliferation and tube formation in vitro and improving healing in a pig model [58]. Perfused, EC-lined microvessels within bioprinted dermis expedite graft survival and host vascular invasion, with constructs containing ECs and pericytes becoming perfused and improving epidermal rete formation within weeks after implantation [59]. Synergistic effects in co-culture demonstrate that pericytes co-printed with ECs stabilize nascent vessels and enhance host vessel infiltration and epidermal maturation [60], while inclusion of adipose-derived microvascular fragments with fibroblasts promoted rapid network formation and epidermal regeneration in vivo [61]. Therapeutic evidence from preclinical studies shows that multilayered grafts printed with dermal ECs/pericytes and an overlying keratinocyte layer formed human EC-lined perfused microvessels that inosculated with host vasculature and were perfused within approximately 4 weeks in immunodeficient mice [62], core–shell peptide–chitosan/dextran with GelMA supported endothelial tubelike structures and roughly doubled in vitro wound closure rates versus controls [63], and GelMA patches carrying a VEGF-mimicking peptide stimulated endothelial proliferation, tube formation, and improved wound repair in a porcine model [64], though there is insufficient evidence for mature, approved clinical products with embedded EC networks in the supplied literature.

Keratinocytes recreate the epidermal barrier through proliferation, stratification, and cornification, with their integration atop dermal bioprinted layers being required for re-epithelialization and barrier restoration [65]. In their role in healing, keratinocytes migrate from wound edges or graft surfaces to form a stratified epidermis that restores barrier function and prevents infection [64,65]. Regarding scaffold interactions, keratinocytes seeded or printed as an epidermal bioink over dermal matrices (collagen, GelMA, gelatin blends) establish confluent basal layers and stratify when supported by appropriate stiffness and basal microstructure [65]. The cellular mechanisms employed by keratinocytes include migration and stratification where keratinocytes establish tight junctions and express differentiation markers (e.g., cytokeratin-14, KRT10) [56], and barrier formation following multilayer maturation in constructs where the dermal bioink provides mechanical support and biochemical cues, with pericyte-containing dermal inks improving keratinocyte maturation and rete ridge formation in vivo. Synergistic effects in co-culture demonstrate that dermal–epidermal cross-talk occurs where fibroblast ECM remodeling and endothelial vascularization create a microenvironment that promotes keratinocyte proliferation and terminal differentiation. Dermal pores and basal dense layers supporting keratinocyte stratification and epidermal thickness gains in vitro [66], while tri-cell hydrogels (fibroblasts, keratinocytes, endothelial progenitors) achieved full re-epithelialization and abundant neovascularization in diabetic and chronic wound rodent models [38]. Therapeutic evidence from preclinical studies shows that bilayer grafts with collagen dermis containing fibroblasts and printed keratinocytes produced a mature stratified epidermis and perfused human microvessels after implantation in mice [62].

Mesenchymal stem cells (MSCs/ADSCs) in printed scaffolds act via differentiation potential and powerful paracrine signaling to reduce inflammation, enhance angiogenesis, and stimulate matrix remodeling, with scaffold mechanics and biochemical additives modulating stem cell secretome and therapeutic potency [67]. In their role in healing, stem cells supply pro-regenerative paracrine factors (VEGF, TGF-β family members), immunomodulation, and potential differentiation toward dermal lineages to accelerate epithelial closure and collagen deposition [68]. Regarding scaffold interactions, encapsulation of ADSCs/MSCs in GelMA-based, gradient-stiffness, or dECM-enriched bioinks preserves viability, enhances migration, and biases secretome profiles, with adipose-derived dECM–GelMA–HAMA scaffolds providing thermo-sensitive printability and a porous, cell-friendly network for hADSCs [69]. The cellular mechanisms employed by stem cells include paracrine angiogenesis where 3D-printed ADSC scaffolds increased VEGF expression and enhanced HUVEC migration and tube formation, linking stem cell paracrine output to neovascularization [67], enhanced proliferation and migration where gradient-stiffness gelatin-alginate scaffolds augmented ADSC proliferation and migration versus uniform scaffolds, improving angiogenesis and wound closure in animal models [70], anti-inflammatory and antioxidant effects where incorporation of bioactive small molecules (e.g., nitric oxide, Salvianolic acid B) with stem cell-laden scaffolds reduced inflammation and oxidative stress and enhanced angiogenesis in burn and diabetic wound models [59,67]. Stem cell-laden constructs promoted earlier vascularization, faster epithelialization, and improved collagen organization in multiple preclinical studies, with VEGF upregulation in ADSCs within 3D scaffolds correlating with increased CD31 + neovessels and faster healing [71]. Synergistic effects in co-culture demonstrate that ADSC microspheres combined with endothelial compartments in coaxial-printed prevascularized skin organoids accelerated vascular closure and activated PI3K-AKT-mTOR signaling to support multistage vessel formation in vivo [72], while MSCs/ADSCs in tri-cell constructs enhance endothelial network maturation and keratinocyte-driven re-epithelialization through paracrine cross-talk. Therapeutic evidence from preclinical studies shows that ADSC-loaded dECM-GelMA-HAMA printed grafts accelerated wound closure, increased blood perfusion, promoted re-epithelialization, and improved collagen deposition in nude mouse full-thickness wound models [73], 3D-ADSCs/NO hydrogel scaffolds for severe burns increased VEGF expression, HUVEC angiogenesis, epithelialization, and collagen deposition in murine models [74], and coaxial-printed prevascularized skin organoids with ADSC microspheres produced abundant neovessels and improved collagen remodeling in large skin defects and implicated PI3K-AKT-mTOR pathway activation in vascular formation in vivo [73].

#### 3.4.2. Growth Factors and Cytokines

Vascular Endothelial Growth Factor (VEGF) is arguably the most critical factor for angiogenesis. It stimulates endothelial cell migration and proliferation. Since VEGF has a very short half-life, researchers often encapsulate it in microspheres or bind it to heparin-based bioinks to ensure a slow, sustained release that prevents the “burst effect” [75].

Platelet-Derived Growth Factor (PDGF) is one of the first factors released during natural injury. In chronic wounds, PDGF levels are often severely depleted. It acts as a potent chemoattractant, “recruiting” fibroblasts and macrophages to the wound site to begin tissue repair. It also stimulates the production of the Extracellular Matrix (ECM). It is frequently used in multimodal bioprinting, where PDGF is printed into the deeper dermal layers to promote structural integrity [76].

Transforming Growth Factor-β (TGF-β) is a “master regulator” that spans multiple phases of healing. It promotes collagen synthesis and helps fibroblasts differentiate into myofibroblasts, which are responsible for wound contraction. In 3D constructs, the concentration must be carefully controlled; excessive TGF-β can lead to hypertrophic scarring rather than functional tissue regeneration [77].

While VEGF and PDGF handle the “foundation” (dermis), Epidermal Growth Factor (EGF) focuses on the “roof” (epidermis). It stimulates re-epithelialization by causing keratinocytes to migrate across the wound surface and divide. EGF is often gradient-printed or top-coated onto the bioprinted scaffold to ensure the surface of the wound seals quickly against pathogens [77].

Specifically, Fibroblast Growth factor (FGF) is a versatile player in tissue engineering. It supports both angiogenesis and the proliferation of various cell types, including fibroblasts and neurocytes. It helps in restoring the mechanical strength of the healed skin, making the treated area less likely to re-ulcerate [76].

Bioprinted constructs can serve as delivery vehicles for growth factors essential to wound healing. Amo et al. demonstrated that bioprinted constructs released VEGF, HGF, IL-8, and MCP-1, with MCP-1 levels increasing up to 70-fold by day 11 [11]. The pro-angiogenic QHREDGS peptide was covalently conjugated to GelMA/HAMA patches to extend its release and improve angiogenic properties [78].

#### 3.4.3. Bioactive Molecules

Incorporated Salvianolic Acid B (Sab) Into Sodium Alginate-Gelatin Scaffolds Using Extrusion Bioprinting, Promoting Diabetic Wound Repair Through Antioxidant and Anti-Inflammatory Mechanisms, Enhancing Granulation Tissue Regeneration and Collagen Deposition [40].

#### 3.4.4. Photosynthetic Microorganisms

In their work Wang et al. incorporated the Oxygenic Photosynthetic Microalga Chlorella Pyrenoidosa Into Bioprinted Scaffolds, Creating Living Photosynthetic Systems That Produced Sustainable Oxygen Under Light Conditions, Alleviating Hypoxia in Diabetic Wounds [21]. Liu et al. Developed Bioprinted Biogenic Hydrogels Inspired by Lichens, Containing Microalgae for Oxygen Supply and Probiotics for Infection Inhibition, Demonstrating Prolonged Biogenetic Oxygen Supply and Enhanced Wound Healing [29].

Photosynthetic microorganisms, particularly Chlorella pyrenoidosa, have emerged as innovative living components in bioprinted scaffolds that address hypoxia through biological oxygen generation. Wang et al. developed in situ microfluidic-assisted 3D bioprinting systems incorporating Chlorella into photocrosslinked bioinks, creating living scaffolds that produced sustained oxygen under light, alleviated hypoxia, and achieved faster wound closure in diabetic models [27]. Liu et al. advanced this with lichen-inspired bioprinted biogenic hydrogels containing both microalgae for oxygen supply and probiotics for infection control, demonstrating rapid tissue repair within 3 days and approximately 90% skin restoration by day 12 in diabetic wounds [33]. The mechanism relies on photosynthesis converting light into dissolved oxygen with delivery efficiency over 100-fold higher than topical gaseous oxygen [3]. Various bioink formulations support photosynthetic activity including GelMA matrices, alginate hydrogels, and microneedle arrays fabricated using microfluidic printing and extrusion bioprinting [25]. These constructs offer multifunctional capabilities beyond oxygenation, including bioelectric signals, growth factor delivery, and infection control, with performance data showing oxygen production for over 30 h and accelerated wound closure [28]. Advantages over hyperbaric oxygen therapy include localized continuous delivery, superior tissue penetration, customizable deposition, and multifunctional responses [29]. Challenges include light-dependent production requiring careful control, maintaining algal viability through proper matrix design, biosafety concerns regarding microbial containment, and complex regulatory pathways, though early human studies showed promising results. Applications are expanding beyond diabetic wound healing to tissue graft survival, programmable therapy with light-controlled switching, and broader regenerative engineering [33].

## 4. Mechanisms of Chronic Wound Healing

Understanding the mechanisms by which bioprinted constructs promote chronic wound healing is essential for rational design and optimization of therapeutic strategies.

### 4.1. Pathophysiology of Chronic Wounds

**Figure 4:**
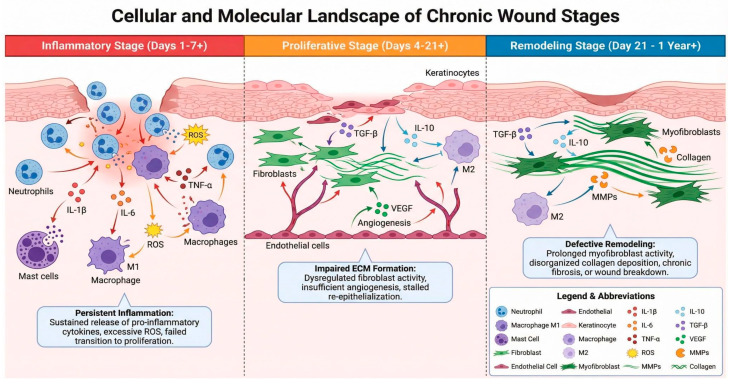
Cellular and molecular landscape of chronic wound stages.

Chronic wounds are characterized by disruptions in the normal wound-healing cascade, which comprises four overlapping phases: hemostasis, inflammation, proliferation, and remodeling [55]. In chronic wounds, these phases are dysregulated, resulting in prolonged inflammation, impaired cellular proliferation, insufficient angiogenesis, and abnormal ECM remodeling [56] (Figure 4).

During the inflammatory phase platelets form a clot and release chemoattractants that recruit neutrophils and mast cells, which release pro-inflammatory cytokines and deploy NETosis to trap and kill pathogens. Tissue-resident macrophages detect PAMPs/DAMPs and amplify the inflammatory response. In impaired wounds this influx is excessive and prolonged, causing tissue damage and stalled progression. In the proliferative wound-healing phase, a provisional fibronectin-rich ECM supports infiltration and fibroblast activity while growth factors stimulate endothelial progenitor cells to drive angiogenesis, restoring oxygen and nutrients to the wound. Monocytes differentiate into inflammatory M1 and pro-regenerative M2 macrophages, with M2s secreting anti-inflammatory cytokines, growth factors, and proteases that promote ECM replacement by collagen and keratinocyte coverage. During the remodeling phase, macrophages, fibroblasts, and myofibroblasts reorganize the provisional matrix into a stronger scar via coordinated MMP and TIMP activity, whereas impaired wounds show poor collagen reorganization, persistent inflammation, and weakened, nonfunctional tissue.

Key impediments to chronic wound healing include persistent inflammation driven by elevated pro-inflammatory cytokines, senescent cell accumulation, impaired growth factor signaling, and the presence of biofilms that resist antibiotics and immune clearance [44]. Some Local factors such as ischemia, infection, and mechanical stress, combined with systemic conditions including diabetes, vascular diseases, and immunodeficiency, further complicate the healing process [45].

Diabetic wounds, in particular, are characterized by hyperglycemia-induced oxidative stress, advanced glycation end-product accumulation, impaired angiogenesis, and neuropathy [46]. The hypoxic microenvironment in diabetic wounds, resulting from microvascular dysfunction, significantly impairs cellular metabolism and tissue regeneration [47].

### 4.2. Angiogenesis and Vascularization

Angiogenesis is essential for supplying oxygen, nutrients, and immune cells to wounds [48]. Chronic wounds, especially diabetic ulcers, show impaired angiogenesis driven by lowered VEGF expression, endothelial dysfunction, and pericyte abnormalities [49]. Apart from standard bioprinted products, adding some specific ingredients like strontium silicate (SS) microcylinders into bioprinted skin substitutes acted as stable angiogenic inducers, markedly increasing microvessel formation and improving graft–host integration [37]. Pro-angiogenic dual-crosslinked collagen bioinks, applied by DPL, improve vascular regeneration in angiogenesis-impaired diabetic wounds [14]. Another adjunct to functionality of bioprinted materials are the photosynthetic scaffolds that generate oxygen via microalgal photosynthesis alleviated hypoxia, raising CD31-positive microvessel density from 5.5 ± 0.1% to 15.6 ± 1.6% and reducing HIF-1α expression from 13.2 ± 1.5% to 1.1 ± 0.2% compared with controls [21]. Bioprinted constructs containing decellularized adipose matrix and plasma released VEGF and HGF, promoting endothelial proliferation, migration, and tube formation [11]. Finally, angiogenesis and tissue repair could be enhanced by 3D-bioprinted peptide patches incorporating the pro-angiogenic QHREDGS sequence [31].

### 4.3. Immunomodulation and Inflammation Control

Biomaterials influence macrophage polarization and inflammation resolution through physical, chemical, and biochemical mechanisms that reprogram macrophages from pro-inflammatory M1 toward anti-inflammatory M2 phenotypes. Physical cues such as scaffold topography and stiffness suppress M1 programs by downregulating NF-κB and JAK-STAT pathways, while surface chemistry variations (alginate M/G ratios, PEGylated chitosan) bias M2 responses [50]. Controlled release of anti-inflammatory cytokines (IL-10, TGF-β), small molecules (protocatechualdehyde, crisaborole), and regulatory RNAs (anti-miR-155) activates key signaling pathways including Jak1/STAT3, TLR2-PI3K/Akt, and STAT6 through epigenetic modulation [41]. Biomaterials enable metabolic transitions from glycolysis-driven M1 toward oxidative phosphorylation-driven M2 metabolism via ROS-scavenging and glucose-consuming systems [29]. Bioprinted constructs integrate staged cytokine release (IL-10 early, then VEGF/PDGF), gene and exosome delivery, antimicrobial activity, and MSC therapy to orchestrate inflammation-to-regeneration transitions [32]. Clinical examples include biogenic hydrogels with microalgae and probiotics achieving approximately 90% skin restoration by day 12, cell-adaptive hydrogels with anisotropic micropores enhancing angiogenesis, core–shell mats releasing crisaborole for M2 induction followed by eugenol for ROS mitigation, and biphasic nanobioinks targeting angiogenesis and inflammation simultaneously [25]. Through coordinated physical/chemical cues, temporal biochemical delivery, metabolic reprogramming, and integration of antimicrobial, angiogenic, and oxygenation functions, biomaterials resolve chronic inflammation by shifting from M1-mediated tissue damage toward M2-mediated repair, angiogenesis, and matrix remodeling.

### 4.4. Extracellular Matrix Remodeling

The extracellular matrix (ECM) provides structural support and biochemical signals that guide cellular behavior and tissue organization [52]. In chronic wounds, however, elevated matrix metalloproteinase (MMP) activity disrupts ECM integrity, causing excessive degradation and impaired collagen deposition [53]. Bioprinted constructs have been shown to promote ECM remodeling through several mechanisms. For example, photosynthetic scaffolds improved ECM synthesis in wounds [21], and double-crosslinked angiogenic patches enhanced ECM remodeling in diabetic wounds [9]. Fibroblasts embedded within bioprinted constructs maintained expression of fibronectin, COL1A1, COL1A2, and COL3A1, reflecting active ECM production [11]. Incorporating Salvianolic acid B into scaffolds boosted granulation tissue formation and collagen deposition via antioxidant and anti-inflammatory effects [40]. Biomimetic multilayer implants made from microfragmented adipose ECM also improved healing by supporting ECM remodeling [54]. Additionally, anisotropic microporous hydrogels encouraged oriented fibroblast alignment and myofibroblast differentiation, promoting ECM remodeling and wound contraction; when co-cultured with human keratinocytes, these constructs formed bilayer skin with tight junctions and increased cytokeratin-14 expression, indicative of proper epithelial differentiation [29].

## 5. Clinical Applications and Outcomes

Recent preclinical and clinical studies have demonstrated the therapeutic efficacy of bioprinted constructs for chronic wound healing, with particular emphasis on diabetic wounds and full-thickness skin defects.

### 5.1. Diabetic Wound Healing

Diabetic wounds are among the most difficult chronic wounds to treat, affecting about 25% of people with diabetes during their lifetime and accounting for most non-traumatic lower-limb amputations [55]. Recent bioprinting approaches show promising results for these wounds. In situ bioprinted photosynthetic scaffolds significantly improved closure in diabetic mice: treated wounds reached 1.1 ± 0.3% relative wound area by day 15 versus 13.0 ± 1.5% in controls, corresponding to roughly 92% closure; the scaffolds also mitigated hypoxia by producing sustained oxygen via microalgal photosynthesis, increasing microvessel density and lowering hypoxia markers [21]. Double-crosslinked angiogenic alginate/chondroitin sulfate patches enhanced angiogenesis and ECM remodeling in diabetic models [9]. To tackle infection risk, levofloxacin-loaded PCL scaffolds provided sustained antibiotic release for four weeks and showed antibacterial activity against common wound pathogens; the 3.0% levofloxacin formulation released 478.23 µg by day 14 [10]. Lichen-inspired biogenic hydrogels incorporating microalgae and probiotics accelerated tissue repair, producing dense vascular networks and restoring about 90% of full-thickness skin structure within 12 days, combining oxygen generation with infection control [41]. Finally, Salvianolic acid B-loaded scaffolds promoted diabetic wound healing via antioxidant and anti-inflammatory actions, improving granulation tissue formation and collagen deposition—beneficial given the elevated oxidative stress in diabetic wounds [40]. Additionally, there is evidence that cutaneous nerve regeneration in diabetic wounds can be improved by using protein-based functional hydrogel, containing zinc ions [77].

### 5.2. Full-Thickness Skin Defects

Full-thickness skin defects, which penetrate the epidermis and dermis into subcutaneous tissue, demand reconstruction of complex skin architecture including multiple cell layers and vascular networks [79]. To address this, Zhang et al. created 3D-bioprinted, human-derived skin organoids made from keratinocytes, fibroblasts, and endothelial cells that developed stromal cores and, when applied to full-thickness wounds in immunodeficient mice, accelerated healing via in situ regeneration, epithelialization, vascularization, and suppression of inflammation; the ability to tailor organoid size and shape improved implantation and graft–host integration [12]. Similarly, Ma et al. used multicellular biomaterial inks containing strontium silicate microcylinders to speed healing in acute and chronic wound models across three animal systems by enhancing graft–host integration and promoting vascularized skin regeneration—a notable advance in combining skin-mimetic structure with vascular function [37]. Zhang et al. also reported that biomimetic multilayer implants composed of microfragmented adipose ECM and cells improved angiogenesis and ECM remodeling in a murine full-thickness wound model [53]. Reviews by Xue et al. summarize evidence that biomimetic constructs incorporating human skin fibroblasts and HUVECs support regeneration of full-thickness defects in pig models [80]. Finally, Jorgensen et al. showed that multicellular bioprinted skin can recreate human-like skin architecture in vivo, marking progress toward clinically relevant skin substitutes by arranging multiple cell types in defined spatial patterns [81].

### 5.3. Comparative Analysis of Bioprinted Constructs

This comparative analysis (Table 1) reveals several key trends. Firstly, extrusion-based bioprinting remains the dominant technique, reflecting its versatility and compatibility with diverse bioinks. Secondly, natural polymers, particularly alginate, gelatin/GelMA, and collagen, are preferentially used due to their biocompatibility and cell-recognition motifs. Thirdly, the incorporation of multiple cell types and bioactive components enhances therapeutic efficacy. Forth, diabetic wound models are the most commonly studied, reflecting the clinical significance of this wound type. Finally, quantitative outcomes demonstrate substantial improvements in wound closure rates, vascularization, and tissue regeneration compared to controls.

## 6. Our Preliminary Clinical Experience with 3D-Bioptining Technology

### 6.1. Overview

The following cases present the author’s original work and include 4 patients which were included in the study upon informed consent. This case series was conducted in accordance with the Declaration of Helsinki. The study protocol was reviewed by the Institutional Review Board (IRB) of Medical University of Plovdiv, which determined its approval. Written informed consent was obtained from all patients. This research received no specific grant from any funding agency in the public, commercial, or not-for-profit sectors. The authors acknowledge the institutional support provided by the Department of Burns, Plastic and Reconstructive Surgery at University Hospital “St. George”, Plovdiv, for infrastructure and time allocation. All authors declare no conflicts of interest regarding the publication of this case series. The study evaluated the clinical efficacy of the 3D Dr. INVIVO Bioprinter, an extrusion-based bioprinting system, in treating refractory chronic wounds. This study provides important real evidence for the clinical translation of 3D bioprinting technology in wound care.

### 6.2. Patient Population and Study Design

Four patients (2 males, 2 females; age range 62–78 years, mean age 70 years) with chronic wounds failing to heal after more than 4 years of both conservative and surgical treatment were enrolled. All patients presented with:

- Wound duration: >4 years of failed treatment;
- Comorbidities: Stage 2 arterial hypertension (well-controlled) in all patients (100%); diabetes mellitus type 2 (well-controlled) in one patient (25%);
- Infection status: Microbiological examination confirmed absence of active infection in all cases;
- Previous treatments: Multiple debridements, advanced wound dressings, negative pressure wound therapy, and failed surgical interventions including skin grafting and local flap reconstruction.

Inclusion Criteria: Documented failure to heal after ≥4 years of treatment; Failed conservative and surgical management; Medical optimization of comorbidities; Patient consent for novel therapeutic approach.

Exclusion Criteria: Active wound infection; Uncontrolled systemic diseases; Malignancy in wound bed; Connective tissue disorder; Psychiatric disorders; Inability to comply with follow-up.

### 6.3. Technology and Treatment Protocol

For the sake of our case series, we used an extrusion-based bioprinting device, 3D Dr. INVIVO Bioprinter, Rokit Healthcare Inc., Seoul, Republic of Korea, which is able to create direct in situ or ex vivo bioprinted materials [79].

**Figure 5:**
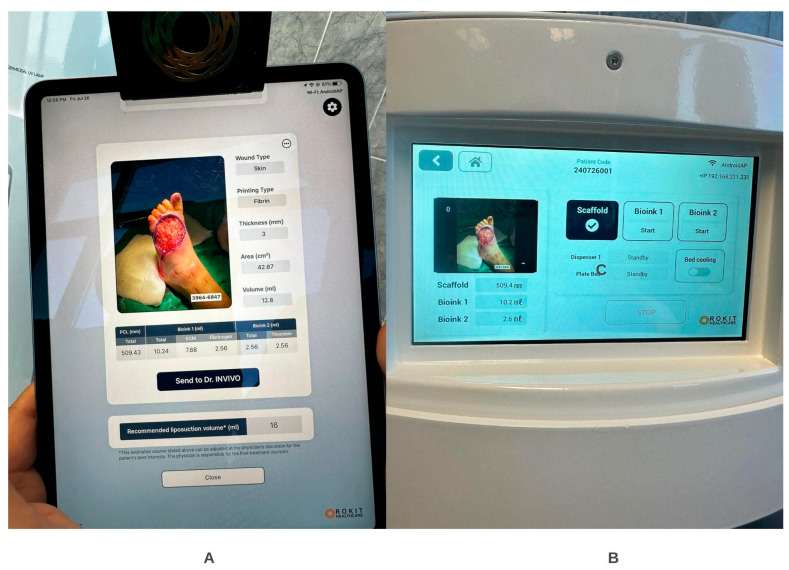
Taking a photo of the wound and setting its parameters into the INVIVO Bioprinter AiD Regen software. (**A**) The debrided and properly prepared wound is pictured with a Dr. In Vivo Software (V2.1.0), which calculates the wound characteristics: size, surface, shape; (**B**) The image is sent to the Bioprinter device which is then set to prepare a scaffold with the proper metrics of the concrete wound; The device create the scaffold using medical Polycaprolactone (PCL), which is melted down by the device, and once it cools down, it gets stiff (Video S1).

**Figure 6:**
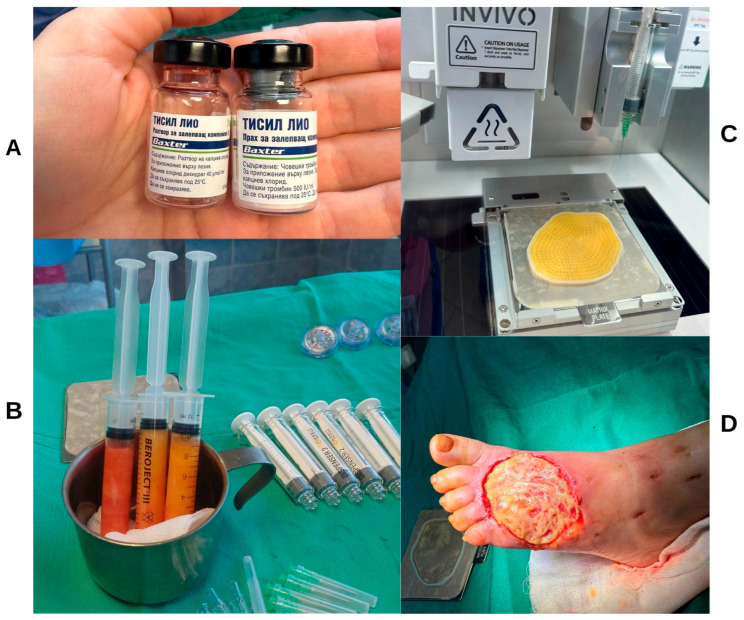
Manufacturing of the 3D-Bioprinted product and its application to the wound. (**A**,**B**) Bioink materials: Autologous nanofat and fibrin glue; The device uses Extrusion-based technology for distributing the nanofat into the scaffold and Inkjet technology for distribution of the fibrin glue (Video S2); (**C**) Final product is detached carefully from the scaffold, removed from the frost plate and placed onto the wound surface; (**D**) Occlusive dressing is then applied and changed weekly.

Treatment Protocol:

1. Comprehensive Wound Assessment and Measurement (Figure 5);
2. Microbiological Sampling and Analysis;
3. Wound Bed Preparation and Debridement, Photo of the Wound;
4. Liposuction, Preparation and Application of 3d Bioprinted Material (Figure 6);
5. Standard Post-Application Wound Care;
6. Weekly follow-up assessments.

The following clinical evolution table [Table 2] presents detailed photographic documentation and outcome data for all four patients treated with the extrusion-based bioprinter. The table tracks wound-healing progression across five time points (preoperative baseline, weeks 2, 5, 8, and 11 postoperative).

### 6.4. Safety Profile

Upon inclusion of the patients in the study, the following parameters were considered:

1. Ensuring adequate infection control;
2. Optimizing comorbid conditions (particularly diabetes);
3. Managing patient expectations, especially in diabetic populations;
4. Appropriate wound bed preparation;
5. Consideration of wound duration and previous treatment failures.

Adverse Events: No serious adverse events reported, No allergic reactions or material rejection, No infections during or after treatment, Well-tolerated by all patients across age spectrum (62–78 years).

Diabetes Mellitus: The only diabetic patient (1/4, 25%) required additional surgical intervention; Three non-diabetic patients (3/3, 100%) achieved complete healing; Despite good glycemic control (HbA1c < 7.0%), diabetes impacted healing outcomes; Bioprinted materials facilitated partial healing, reducing defect size, which were subsequently grafted.

### 6.5. Clinical Outcomes

Overall Healing Success: Complete epithelialization at 11 weeks: 3/4 patients (75%) Incomplete epithelialization requiring intervention: 1/4 patients (25%) [Table 2].

## 7. Comparison with Conventional Approaches

While conventional wound dressings serve primarily as passive or semi-active barriers designed to manage the wound microenvironment—controlling moisture, preventing infection, and absorbing exudate without contributing structural biology—they fundamentally lack the capacity to replace lost tissue or restore complex skin architecture. In contrast, skin grafting, specifically the autograft, remains the clinical “gold standard” because it immediately transplants living, functional tissue to restore barrier integrity; however, this approach is inherently limited by the availability of healthy donor sites, the creation of secondary wounds that cause additional pain and scarring, and the inability to cover extensive burns or chronic ulcers effectively when donor skin is scarce [82].

3D bioprinting emerges as a revolutionary alternative that transcends these limitations by shifting the strategy from simple coverage or transplantation to active fabrication and regeneration. By utilizing sophisticated computer-aided design to deposit layer-by-layer bioinks laden with patient-specific cells (such as keratinocytes, fibroblasts, and potentially endothelial cells), bioprinting aims to recreate the precise, multi-layered anatomy of natural skin, including the potential for pre-vascularized networks that accelerate integration—something neither dressings nor standard grafts can manufacture on demand. Although this technology promises to eliminate donor site morbidity and enable the treatment of complex, deep-tissue defects with reduced scarring, it is currently hindered by high costs, slow fabrication speeds, and significant regulatory hurdles regarding cell sourcing and standardization, whereas dressings and grafts benefit from decades of established clinical protocols and immediate scalability [79].

Traditional approaches like skin grafting (split-thickness or full-thickness), local or regional flaps and free tissue transfer are associated with graft failure risk, donor site morbidity and additional surgeries.

3D Bioprinting shows the following advantages: 1. Minimally invasive procedure: No donor site required; 2. Customization: Materials tailored to specific wound characteristics; 3. Biological activity: Incorporation of cells and growth factors; 4. Reduced morbidity: Avoids additional surgical sites; 5. Success in refractory cases: 75% complete healing in patients who failed all conventional therapies.

## 8. Limitations and Future Research Directions

Future Research Directions:

Randomized controlled trials: Direct comparison with standard surgical approaches;

Optimization studies: Investigating optimal bioink compositions, cell types, and printing parameters;

Biomarker analysis: Identifying predictors of treatment response;

Diabetic wound protocols: Developing specialized approaches for diabetic patients;

Long-term follow-up: Assessing healing durability and recurrence rates;

Cost-effectiveness analysis: Economic evaluation compared to conventional treatments;

Expanded indications: Application in burns, surgical wounds, and other health conditions.

### 8.1. Technical Challenges

Vascularization: The formation of functional vascular networks within bioprinted constructs remains a critical challenge [64]. While recent studies have demonstrated enhanced angiogenesis through incorporation of pro-angiogenic factors and cells [14,35], the creation of perfusable vascular networks capable of immediate integration with host vasculature requires further innovation. Strategies under investigation include prevascularization in bioreactors, incorporation of endothelial cells and pericytes in defined spatial arrangements, and the use of sacrificial materials to create vascular channels [83].

#### Challenges in Vascularization of Large Bioprinted Constructs

Vascularization represents the critical challenge limiting clinical translation of large bioprinted constructs, as tissue viability beyond 100–200 μm depends on functional blood vessel networks delivering oxygen and nutrients while removing metabolic waste. Without perfusable vasculature, cells in thick construct interiors rapidly experience hypoxia and necrosis. The challenge is compounded by the need for rapid anastomosis between bioprinted vasculature and host circulation upon implantation. To address these limitations, the field has developed complementary strategies. Prevascularization involves creating endothelial microvessel networks within constructs prior to implantation through co-culture of endothelial cells with supporting cells (MSCs, pericytes); these pre-formed networks mature in vitro and rapidly anastomose with host vasculature, dramatically improving construct survival [64]. Bioprinted fibrin constructs containing HUVECs co-cultured with MSCs demonstrated enhanced vascularization and increased bone formation in rat femoral defects [63]. Coaxial bioprinting uses core-sheath nozzle systems to create hollow tubular structures with endothelialized lumens, producing centimeter-scale vascularized constructs maintaining high viability over 14–20 days [21]. Sacrificial templating prints fugitive inks that form channel networks and are subsequently removed to leave hollow, perfusable conduits, creating branched hierarchical architectures [23]. Engineered customizable microvessels fabricated through coaxial microfluidic extrusion represent modular vascular building blocks that sprout daughter vessels and anastomose with host vasculature in vivo [23]. Growth factor delivery strategies provide biochemical cues: spatially patterned VEGF drives vessel sprouting, bFGF stimulates endothelial proliferation, and PDGF-BB recruits mural cells for stabilization [64]. Dual-factor constructs positioning VEGF centrally and BMP-2 peripherally demonstrated enhanced microvessel formation and accelerated bone regeneration. Design optimization reveals that spatial placement significantly impacts outcomes: distal microvascular patterns showed 2-fold improved neo-vascularization, and combining microvascular patterns with angiogenic cell patterns produced 1.5–1.9-fold higher neo-vessel volume [84]. Microfluidic architectures enable hierarchical vascular networks with vessel diameters from capillary-scale to arteriole-scale; the VesselNet platform enabled direct surgical anastomosis and demonstrated host perfusion within two weeks [84]. Dynamic perfusion culture using bioreactors applies physiological flow and shear stress, promoting endothelial alignment and vessel maturation. Quantitative outcomes demonstrate efficacy: laser-assisted bioprinting increased vascularization rates by 203–355% and bone regeneration by 294–602% [84]; coaxial constructs maintained cell viability of 492 ± 18.8% at 14 days [18]. Through these complementary strategies—prevascularization, coaxial and sacrificial printing, growth factor delivery, microfluidic designs, and dynamic culture—the field is progressively overcoming the vascularization bottleneck toward clinically viable, centimeter-scale, perfused tissue constructs.

Printing Resolution and Cell Viability: A fundamental trade-off exists between printing resolution and cell viability [18]. High-resolution printing requires small nozzle diameters and high shear forces, which can compromise cell viability. Conversely, conditions that maximize cell viability often result in lower resolution and structural fidelity [19]. DLP bioprinting offers improved resolution but introduces concerns regarding phototoxicity from UV exposure [59]. Future developments in bioprinting hardware, bioink formulations, and crosslinking strategies are needed to optimize this balance.

Mechanical Properties: Bioprinted constructs must possess mechanical properties appropriate for wound-healing applications, providing sufficient structural integrity while remaining flexible and conformable to wound beds [60]. Glover et al. demonstrated that scaffold design significantly influences mechanical properties, with square designs exhibiting lower tensile stiffness (25.30 N/mm) suitable for flexible wound dressings [10]. The integration of synthetic polymers such as PCL can enhance mechanical strength, but may compromise cellular infiltration and remodeling [61].

### 8.2. Emerging Innovations

Despite rapid technical progress, translating 3D bioprinting from bench to bedside faces several interrelated challenges that must be addressed in coming years. Key scientific barriers include scalable vascularization and innervation of thick tissues, long-term functional maturation and integration of printed constructs, and standardization of bioinks and cell sources to ensure reproducibility and viability across platforms. Translationally, robust GMP-compliant biomanufacturing workflows, rigorous preclinical models that predict human outcomes, and cost-effective scaling are required to move beyond bespoke academic demonstrations to commercial clinical products. Regulatory pathways remain nascent: agencies will need clear frameworks for classification (device, biologic, combination product), validated assays for potency, sterility, and biosafety, and guidance on acceptable analytical and clinical endpoints for heterogeneous, patient-specific constructs. Anticipated near-term developments include improved multi-material and multimodal printers, integrated imaging/biomonitoring and AI-driven design/quality control, maturation strategies (bioreactors, mechanical/electrical conditioning), and expanded use of allogeneic or iPSC-derived cell banks—all of which should accelerate clinical translation if accompanied by coordinated standards, reimbursement models, and ethical oversight [85].

Integration with Advanced Technologies: The integration of bioprinting with artificial intelligence and machine learning enables optimization of bioink formulations, printing parameters, and construct designs based on patient-specific data. Computational modeling can predict construct behavior and guide rational design [72].

Smart and Responsive Bioinks: The development of bioinks that respond to environmental stimuli, such as pH, temperature, or enzymatic activity, enables dynamic modulation of construct properties in response to wound-healing progression [86]. Stimuli-responsive drug release systems can provide temporal control over therapeutic agent delivery [74].

Bioprinting with Stem Cells and Induced Pluripotent Stem Cells: The use of stem cells and induced pluripotent stem cells (iPSCs) offers the potential for patient-specific, autologous constructs with enhanced regenerative capacity [87]. However, challenges related to differentiation control, tumorigenicity risk, and regulatory approval must be addressed [76].

Hybrid Approaches: Combining bioprinting with other advanced technologies, such as electrospinning, microfluidics, and organ-on-a-chip systems, enables the creation of hierarchical structures that more closely mimic native tissue complexity [88]. Weaver et al. demonstrated the potential of combining microfluidics with coaxial bioprinting for diabetic wound-healing dressings [89].

In Situ Bioprinting Advancement: Further development of portable, handheld bioprinting devices for in situ application in clinical settings represents a promising direction [79]. These devices must be user-friendly, cost-effective, and capable of real-time wound imaging and construct customization [90].

### 8.3. Scalability Issues and Manufacturing Challenges Associated with Clinical Translation of Bioprinted Tissues

The transition of bioprinting from laboratory prototypes to industrial-scale clinical products is currently hindered by four primary pillars of constraints: Scalability, Manufacturing, and Economic/Technical Logistics [18].

#### 8.3.1. Scalability and Process Bottlenecks

Current bioprinting is limited by slow, serial production and lengthy post-print tissue maturation. High-cell-density constructs require massive cell expansion, leading to long lead times and small batch sizes. Moving to industrial throughput requires a shift toward parallelized nozzle arrays, robotic handling, and integrated “unit operations” to coordinate deposition and culture at scale. Translating laboratory-scale bioprinting to clinical-scale manufacturing presents significant challenges related to reproducibility, quality control, and cost-effectiveness [62]. Automated bioprinting systems with integrated quality monitoring, standardized bioink formulations, and good manufacturing practice (GMP)-compliant production facilities are essential for clinical translation [63].

#### 8.3.2. Manufacturing and Quality Control

Shifting to GMP (Good Manufacturing Practice) standards requires eliminating manual variability. The field lacks universal standards for bioinks and print parameters, leading to significant batch heterogeneity. Solutions involve: utilizing closed-environment robotic platforms (like the ReBiA system) to ensure reproducibility; implementing real-time sensors and automated release assays for cell viability and function; developing digital supervisory systems to track cells and reagents throughout the lifecycle.

#### 8.3.3. Supply Chain and Economic Risks

Economic viability is threatened by high costs associated with clinical-grade materials, cleanroom operations, and cold-chain logistics. Bioinks and cells have limited shelf lives and strict temperature requirements, complicating distribution. Organizations like BioFabUSA and the HITS-Bio group are addressing these via reducing per-unit costs through high-speed, multi-array printing and establishing validated supplier networks and robust storage protocols to maintain sterility and function during transport [19].

Regulatory Approval: Bioprinted constructs incorporating living cells are classified as advanced therapy medicinal products (ATMPs) in Europe and combination products in the United States, requiring extensive preclinical and clinical testing to demonstrate safety and efficacy [64]. The regulatory pathway for personalized bioprinted constructs, which may vary in composition for each patient, presents unique challenges [65]. The regulatory landscape for living, bioprinted constructs is complex and lacks a “one-size-fits-all” framework. Developers face rigorous requirements for sterility, potency assays, and clinical evidence. Success depends on early engagement with agencies and the creation of exhaustive documentation, including Design History Files and validated manufacturing master records [85].

Clinical Validation: While preclinical studies have demonstrated promising outcomes, clinical trials are needed to validate the safety and efficacy of bioprinted constructs in human patients [66]. Abuhamad et al.’s scoping review noted that while 3D-printed bioinks offer transformative potential for chronic wound care, further research is necessary to optimize bioink formulations and printing parameters for clinical application [38].

Cost-Effectiveness: The high costs associated with bioprinting equipment, bioink materials, and skilled personnel may limit accessibility [69]. Economic analyses comparing bioprinted constructs to conventional treatments, considering factors such as healing time, hospitalization costs, and quality of life improvements, are needed to justify clinical adoption [68].

Standardization: The lack of standardized protocols for bioink preparation, printing parameters, and construct characterization hinders reproducibility and comparison across studies [69]. The development of consensus standards through collaborative efforts among researchers, clinicians, and regulatory agencies is essential [51].

## 9. Conclusions

Three-dimensional bioprinting has emerged as a transformative technology for chronic wound treatment, offering unprecedented capabilities for fabricating patient-specific, biomimetic tissue constructs that address the complex pathophysiology underlying chronic wounds. Recent advances (2020–2026) have demonstrated that bioprinted constructs incorporating living cells, bioactive molecules, and innovative biomaterials can significantly accelerate wound closure, enhance vascularization, modulate inflammation, and restore functional skin architecture. Key innovations include in situ bioprinting systems that enable direct deposition onto wound beds, photosynthetic scaffolds that alleviate hypoxia through biological oxygen generation, pro-angiogenic bioinks that enhance vascularization in angiogenesis-impaired wounds, and immunomodulatory constructs that regulate inflammatory responses. Diverse bioprinting techniques—including extrusion-based, DLP, coaxial, and microfluidic approaches—have been successfully employed, with extrusion-based bioprinting remaining the most widely adopted due to its versatility and compatibility with high-viscosity bioinks.

Natural polymers such as alginate, gelatin/GelMA, collagen, and hyaluronic acid are preferentially used due to their biocompatibility and cell-recognition motifs, while synthetic polymers like PCL provide mechanical reinforcement. Composite and hybrid bioinks that combine multiple materials and incorporate cells, growth factors, and bioactive molecules demonstrate superior therapeutic efficacy compared to single-component systems. Preclinical studies have demonstrated remarkable outcomes, with some bioprinted constructs achieving over 90% wound closure within 15 days in diabetic wound models and restoring approximately 90% of skin structure within 12 days. Enhanced angiogenesis, with up to 3-fold increases in microvessel density, and substantial reductions in hypoxic and inflammatory markers have been consistently observed.

Building upon these promising preclinical foundations, future research priorities must pivot toward the recreation of full skin complexity and the establishment of robust manufacturing standards. A critical next step is the engineering of hierarchically organized vascular networks—moving beyond simple capillary sprouting to develop macro-vasculature capable of immediate surgical anastomosis, which is essential for the survival of thick, full-thickness grafts. Furthermore, research must focus on the incorporation of skin appendages, such as hair follicles, sweat glands, and nerve endings; the inclusion of hair follicles is particularly vital as they serve as reservoirs for epidermal stem cells that drive long-term regeneration. Another priority lies in the development of “4D bioprinting” strategies, utilizing smart bioinks that can dynamically reshape or release therapeutics in response to the changing pH or enzymatic environment of a healing wound. Finally, the integration of real-time monitoring sensors within printed constructs to track infection and healing progress represents a crucial frontier for smart wound management.

In terms of clinical applications, the trajectory of bioprinting technology points toward several distinct therapeutic avenues. The primary application remains the treatment of recalcitrant diabetic foot ulcers and venous leg ulcers, where personalized constructs derived from autologous induced pluripotent stem cells (iPSCs) could overcome the limitations of donor site scarcity and immune rejection. Beyond chronic wounds, high-throughput in situ bioprinting holds immense potential for acute burn care, allowing for the rapid coverage of massive Total Body Surface Area (TBSA) burns where traditional grafting is impossible. Additionally, the precision of bioprinting opens new doors for treating genodermatoses (such as Epidermolysis Bullosa) by printing gene-corrected autologous cells, as well as for esthetic and reconstructive surgery, where “scar-free” healing is the ultimate goal.

However, significant challenges remain. Vascularization of thick constructs, optimization of the trade-off between printing resolution and cell viability, achievement of appropriate mechanical properties, scalability for clinical manufacturing, regulatory approval pathways, clinical validation through human trials, cost-effectiveness, and standardization of protocols all require continued research and innovation. The convergence of advances in bioprinting technologies, biomaterial science, cell biology, and regenerative medicine principles positions 3D bioprinting as a promising solution for chronic wound management. Continued multidisciplinary collaboration among engineers, materials scientists, biologists, clinicians, and regulatory experts is essential to translate these promising preclinical findings into clinical reality and improve outcomes for the millions of patients suffering from chronic wounds worldwide.

## Figures and Tables

**Table 1:** Comparative Analysis of Bioprinted Constructs for Chronic Wound Healing.

Authors Years	Bionk Composition	Cell Types	Growth Factors	Bioprinting Technique	Wound Model	Key Outcomes
Freeman et al. 2020 [72]	Nanoparticle-functionalized bioinks enabling spatial patterns of proteins (GF-loaded nanoparticles)	Acellular implants in some conditions	VEGF and BMP-2 spatial patterns printed	Extrusion bioprinting of nanoparticle-functionalized inks	In vivo large bone defect model (demonstrates spatiotemporal GF patterning)	Spatial VEGF patterns increased vessel invasion and angiogenesis versus homogeneous loading, showing controlled angiogenesis via printed GF patterns [1].
Baltazar et al. 2020 [62]	Rat tail type I collagen dermal bioink; second epidermal bioink for keratinocytes	Human foreskin dermal fibroblasts, cord blood-derived endothelial colony-forming cells, placental pericytes, keratinocytes	None reported as exogenous GFs	3D bioprinting to create multilayered grafts (layered collagen dermis + keratinocyte epidermis)	Implantation on dorsum of immunodeficient mice	Printed ECs and PCs self-assembled into perfused human microvessels that inosculated with host vessels by 4 weeks; improved epidermal rete and graft perfusion [2].
Huyan et al. 2020 [61]	Gelatin-sodium alginate composite hydrogel dermis; gel-based epithelium	Human dermal fibroblasts, human microvascular endothelial cells, human keratinocytes	No exogenous GF reported	Extrusion 3D printing to form bilayer skin graft	Full-thickness dorsal wounds in nude mice	~10% improved wound contraction versus control; transplanted cells survived, promoted angiogenesis, and contributed to dermal/epidermal repair.
Jimi et al. 2020 [73]	Chitosan-based cryogel (sequentially loaded with factors)—controlled release system, not conventional bioink	Not cell-laden (growth factor/cytokine delivery scaffold)	Sequential IL-10, TGF-β (early), then VEGF and FGF (late)	Cryogel fabrication and topical application (not nozzle bioprinting)	Murine internal splint wound model	Sequential delivery accelerated wound closure (significant reduction by day 7 and down to ~10% area by day 10), enhanced granulation and functional neovascularization.
Jang et al. 2021 [76]	Gelatin methacrylate (GelMA) hydrogel incorporating a VEGF-mimicking peptide	In vitro assays with NIH-3T3 fibroblasts and HUVECs (patches acellular in vivo)	VEGF-mimicking peptide incorporated into GelMA	Extrusion 3D printing of hydrogel patch	Full-thickness pig skin wound model	Printed GelMA-VEGF peptide patches promoted endothelial tube formation in vitro and improved wound healing and angiogenesis in pig skin wounds.
Siebert et al. 2021 [74]	Hydrogel matrices incorporating tetrapodal ZnO for light-triggered GF release	In vitro endothelial cell assays reported	VEGF loaded and released on light trigger	Bioink deposition with light-responsive components (printed hydrogel scaffolds)	In vitro angiogenesis assays and material characterization	Light-triggered VEGF release at therapeutically relevant doses promoted endothelial migration and tubular formation while avoiding overdose risk.
Wu et al. 2022 [75]	Bioprintable biodegradable polyurethane (PU) blended with gelatin hydrogel	Tri-cell-laden: fibroblasts, keratinocytes, endothelial progenitor cells (EPCs)	No exogenous GF reported	Planar and curvilinear extrusion bioprinting modules for shape-customized constructs	Normal and diabetic rat large/irregular skin wounds	Full re-epithelialization and dermal repair with abundant neovascularization and collagen deposition by 28 days in both normal and diabetic rats.
Baltazar et al. 2022 (xeno-free) [67]	Xeno-free bioink of human collagen I and fibronectin layered on PGA mesh	Xeno-free human endothelial cells, fibroblasts, pericytes; keratinocytes seeded to form epidermis	No exogenous GF reported	3D bioprinting to deposit dermal bioink and seeded epidermis	Implantation on dorsum of immunodeficient mice	Keratinocytes formed stratified epidermis; human ECs and pericytes formed perfused human microvessels within 2 weeks, preventing graft necrosis.
Nuutila et al. 2022 [82]	Adhesive scaffold matrix formulated for point-of-care printing delivering dissolved GFs	Not cell-laden (GF-eluting scaffold delivered in situ)	VEGF used as active GF	In vivo printing (handheld in situ deposition) of adhesive GF-eluting scaffolds	In vivo wound models (rodent), in situ printed onto wounds	In vivo printed VEGF-eluting scaffolds enhanced endothelial migration and improved histologic healing metrics versus controls.
Wang et al. 2023 [60]	Layered GelMA/bacterial nano-cellulose (BNC) formulations (e.g., 10% GelMA/0.3% BNC dermis; 10% GelMA/1.5% BNC basal)	Human dermal fibroblasts and keratinocytes to form heterogeneous tissue-engineered skin	No exogenous GF reported	Extrusion 3D bioprinting with tuned bioink formulations for layered heterogeneity	Full-thickness wound-healing experiments in vivo (animal model)	HTS supported epidermal stratification (epidermal thickness ~80 μm at 14 days), improved granulation, ECM remodeling, and hair-follicle–like structures in vivo.
Ma et al. 2023 [65]	Gradient-stiffness Gelatin-Alginate hydrogel (secondary crosslinking on Ca^2+^ substrate)	Adipose-derived stem cells (ADSCs) encapsulated	No exogenous GF; ADSC paracrine factors implicated	Extrusion bioprinting to produce gradient stiffness scaffolds	Animal full-thickness wound model	Gradient scaffolds enhanced ADSC proliferation/migration, increased paracrine angiogenic signaling, and improved angiogenesis and wound healing versus uniform scaffolds].
Liao et al. 2023 [9]	Double-crosslinked alginate/chondroitin sulfate patch with photocovalent crosslinked VEGF	Acellular patch used to recruit host cells in diabetic wounds	VEGF photocovalently immobilized/released	3D bioprinted patches with photocrosslinking	Diabetic wound model in vivo	Patch promoted diabetic wound healing with enhanced angiogenesis and tissue repair in vivo.
Fu et al. 2026 [26]	Adipose-derived decellularized ECM (dECM) pre-gel blended with GelMA and HAMA	Human adipose-derived stem cells (hADSCs) loaded into printed scaffold	No exogenous GF reported (hADSC paracrine activity)	3D printing of dECM-GelMA-HAMA constructs	Full-thickness dorsal wounds in nude mice	3D-printed constructs accelerated wound closure, reduced inflammation, increased blood perfusion, promoted re-epithelialization, angiogenesis, and collagen deposition.
Li et al. 2024 [69]	Heparin-functionalized bioink (GH/HepMA): HA 0.3% + GelMA 10% + HepMA 0.5% for sustained GF binding	Human dermal fibroblasts and HUVECs used in constructs	VEGF loaded for 21-day sustained release	Extrusion 3D bioprinting of covalently crosslinked heparinized ink	In vitro multicellular dermal constructs and vascular network assays	GH/HepMA enabled 21-day sustained VEGF release, promoted HUVEC proliferation/migration and formation of mature capillary-like networks and enhanced collagen I/III deposition in printed dermal constructs.
Pajooh et al. 2024 [79]	Bilayer scaffold: 3D-printed dextran-VEGF upper layer + electrospun gelatin-keratin bottom layer	Host cell recruitment (acellular scaffold)	VEGF incorporated into printed upper layer	Combined 3D printing and electrospinning fabrication	CAM assay and in vivo animal skin models	VEGF-loaded bilayer scaffold showed highest angiogenic potential in CAM and the best wound-healing rate within 14 days in animal tests.
Shi et al. 2024 [59]	GelMA/sodium alginate (SA) therm o/ion/photo-crosslinked hydrogel with shear-oriented PEO filler producing anisotropic micropores	Human fibroblasts in dermal layer; co-culture with human keratinocytes for bilayer	No exogenous GF reported	Extrusion bioprinting with oriented filler to produce anisotropic pores	Full-thickness wound model in vivo	Anisotropic micropores guided fibroblast alignment, promoted myofibroblast transition, mitigated inflammation, stimulated angiogenesis and ECM remodeling, accelerating full-thickness wound closure.
Zhang et al. 2024 [12]	Selected biocompatible bioink (suitable for organoid spheres) with dual-photo crosslinking to stabilize organoids	Skin organoid spheres comprising human keratinocytes, fibroblasts, endothelial cells	No exogenous GF reported; organoid paracrine signaling	Extrusion bioprinting combined with dual-photo crosslinking to print organoid units	Customized full-thickness defects in immunodeficient mice	3D-printed human-derived skin organoids accelerated wound closure via in situ regeneration: enhanced epithelialization, vascularization, reduced inflammation, and ECM remodeling.
Rana et al. 2024 [80]	Aptamer-based programmable bioinks that sequester and release VEGF on demand	HUVECs and endothelial network formation assays in printed constructs	VEGF sequestered via aptamers and released by complementary sequence	Extrusion/in-bath bioprinting of spatially resolved aptamer inks with temporal trigger	In vitro vascular morphogenesis in 3D printed constructs	Programmable bioinks enabled spatially confined VEGF presentation and CS-triggered release, improving vessel density, branching, and average vessel length compared to non-triggered controls.
Priya et al. 2024 [83]	VascuBiomatrixTM bioink with PODS^®^ encapsulated growth factors for controlled GF delivery in printed vascular grafts	ADSCs differentiated towards endothelial and smooth muscle lineages in/adherent to printed graft	VEGF-165 PODS^®^ and TGF-β1 PODS^®^ to drive differentiation	3D bioprinting of bilayer vascular structures	Vascular graft development and CAM assay/ex ovo angiogenesis tests	Printed vessels with ADSC-derived endothelium and SMCs showed physiological properties, ECM deposition, hemocompatibility, and angiogenic potential in CAM assays.
Kang et al. 2025 [64]	Patient-derived decellularized ECM (pddECM) blended bioink combined with keratin-alginate (KA) bioink layers	Autologous dermal fibroblasts and keratinocytes used in constructs	No exogenous GF reported	Extrusion 3D bioprinting of layered pddECM + KA constructs	In vivo wound-healing tests (animal models)	pddECM supported HDF viability and collagen I production; GelMA + pddECM scaffolds accelerated wound closure, improved angiogenesis, ECM remodeling, and reduced pro-inflammatory cytokines.
Wang et al. 2026 [63]	Coaxial system: inner sacrificial temperature-responsive material forming hollow vascular channels + outer biomimetic hydrogel containing ADSC microspheres and fibroblasts	Endothelial cells in inner channel; outer phase contained ADSC microspheres and skin fibroblasts	ADSC microspheres activated PI3K-AKT-mTOR signaling leading to pro-angiogenic paracrine effects (no exogenous GF)	One-step coaxial 3D printing (PV-XOM) to fabricate pre-vascularized organoid modules	In vitro vascular closure assays and in vivo large skin defect implantation	Rapid vascular closure and maturation in vitro; in vivo formed abundant neovessels, accelerated wound closure and improved collagen remodeling versus controls.

**Table 2:** Clinical Evolution of 3D Bioprinting Treatment—All Four Patients.

Patient Information	Preoperative (Baseline)	Week 2 Postoperative	Week 5 Postoperative	Week 8 Postoperative	Week 11 Postoperative
PATIENT 1 67-year-old Male Hypertension (controlled) Wound Duration: 4.5 years	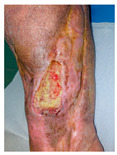 Chronic wound (4.5 years) Failed multiple debridements Failed NPWT Non-healing, stalled epithelialization Infection: Negative Minimal granulation tissue	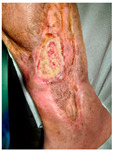 Post-bioprinting (2 weeks) Initial material integration Early cellular migration No infection/rejection Standard wound care maintained	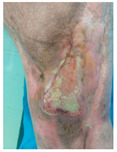 Mid-treatment (5 weeks) Progressive epithelialization Increased granulation tissue Wound contraction beginning Scaffold integration ongoing No adverse events	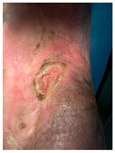 Advanced healing (8 weeks) Significant size reduction Robust epithelialization Healthy granulation tissue Continued remodeling Patient tolerating well	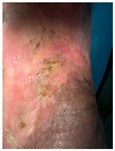 COMPLETE EPITHELIALIZATION 100% wound closure achieved Mature epithelium present No residual defect Successful outcome No complications
PATIENT 2 62-year-old Female Hypertension (controlled) Wound Duration: 4.2 years	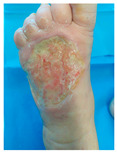 Chronic wound (4.2 years) Failed conservative management Failed surgical closure attempt Persistent non-healing defect Infection: Negative Previous surgery unsuccessful	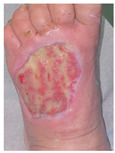 Post-bioprinting (2 weeks) Material adherence confirmed Initial wound bed response Early cellular infiltration No material rejection Well-tolerated treatment	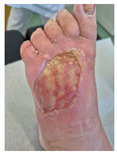 Mid-treatment (5 weeks) Progressive healing response Peripheral epithelialization Improved wound bed quality Bioink scaffold supporting regeneration Continued positive response	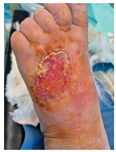 Advanced healing (8 weeks) Marked wound contraction Accelerated epithelialization Healthy vascular tissue formation Success where previous surgery failed No complications	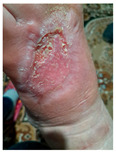 COMPLETE EPITHELIALIZATION 100% wound closure achieved Successful where surgery failed Mature epithelial layer No residual defect
PATIENT 3 78-year-old Male Hypertension (controlled) Wound Duration: 5.1 years (Longest duration) (Oldest patient)	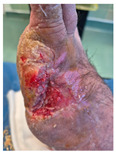 Chronic wound (5.1 years) LONGEST DURATION IN SERIES Extended conservative management failed Failed flap coverage attempt Severely chronic, non-healing Infection: Negative Oldest patient (78 years)	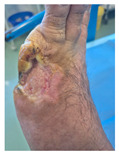 Post-bioprinting (2 weeks) Successful material integration Initial healing in elderly patient Wound bed improvement noted No age-related complications Good tolerance despite age	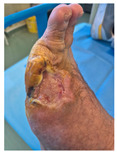 Mid-treatment (5 weeks) Encouraging healing progression Epithelialization advancing despite age Granulation tissue formation Positive geriatric response No adverse events	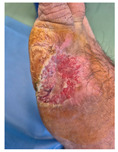 Advanced healing (8 weeks) Continued wound size reduction Strong epithelialization despite prolonged duration Healthy tissue regeneration Efficacy in elderly demonstrated	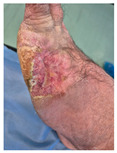 COMPLETE EPITHELIALIZATION 100% wound closure achieved Successful in 78-year-old patient Longest duration (5.1 years) treated successfully Demonstrates efficacy across age spectrum No complications
PATIENT 4 74-year-old Male Hypertension (controlled) + DIABETES MELLITUS Type 2 (HbA1c < 7.0%, controlled) Wound Duration: 4.8 years (ONLY DIABETIC PATIENT)	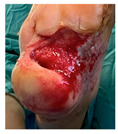 Chronic diabetic wound (4.8 years) Failed comprehensive diabetic care Failed advanced biologics treatment Chronic diabetic wound characteristics Infection: Negative Good glycemic control maintained	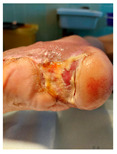 Post-bioprinting (2 weeks) Initial integration in diabetic wound bed Early response observed Diabetic healing challenges evident No infection/rejection Slower initial response vs. non-diabetic	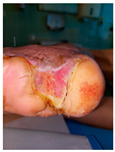 Mid-treatment (5 weeks) Partial healing response Wound size reduction observed Epithelialization slower than non-diabetic Diabetes impacting healing kinetics Bioprinted material facilitating partial closure	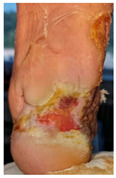 Advanced healing (8 weeks) Continued partial improvement Significant wound size reduction Incomplete epithelialization Residual defect remaining Diabetes limiting complete closure despite good control	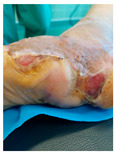 INCOMPLETE EPITHELIALIZATION Partial wound closure achieved Residual defect present REQUIRED SKIN GRAFTING Bioprinting reduced defect size significantly Facilitated subsequent surgical intervention Demonstrates diabetes impact despite control

## Data Availability

The original contributions presented in this study are included in the article. Further inquiries can be directed to the corresponding author.

## Supplementary Materials

The following supporting information can be downloaded at: https://drive.google.com/file/d/1eapKW1iYn6dVQ9nsG4gn4YnhB5I53zpJ/view?usp=drive_web and https://drive.google.com/file/d/1ODftGI4D0WUuAX8H6fLxTqo_2MmnYSbw/view?usp=drive_web (accessed on 13 February 2026).
